# The Impact of Parental Support on Adherence to Therapist-Assisted Internet-Delivered Acceptance and Commitment Therapy in Primary Care for Adolescents With Anxiety: Naturalistic 12-Month Follow-Up Study

**DOI:** 10.2196/59489

**Published:** 2025-01-03

**Authors:** Anna Larsson, Sandra Weineland, Linnea Nissling, Josefine L Lilja

**Affiliations:** 1 General Practice/Family Medicine, School of Public Health and Community Medicine, Institute of Medicine Sahlgrenska Academy University of Gothenburg Gothenburg Sweden; 2 Research, Education Development & Innovation, Primary Health Care Region Västra Götaland Borås Sweden; 3 Department of Psychology University of Gothenburg Gothenburg Sweden; 4 Department of Psychology Faculty of Health and Life Sciences Linnaeus University Växjö Sweden

**Keywords:** adolescents, parental support, anxiety, depression, primary care, mental health, ACT, acceptance and commitment therapy, iACT, internet-delivered acceptance and commitment therapy

## Abstract

**Background:**

Mental health problems among adolescents are increasing, and internet-delivered acceptance and commitment therapy (iACT) constitutes a possible way to improve access to care while reducing costs. Nevertheless, few studies have investigated iACT for adolescents in regular primary care nor the role of parental support.

**Objective:**

This is an exploratory evaluation investigating iACT, with or without parental support, for adolescents. The aims were to examine treatment adherence, symptoms of anxiety and depression, psychological flexibility, and overall functioning.

**Methods:**

Adolescents with anxiety were recruited within the regular primary care patient flow during the implementation phase of therapist-assisted iACT for adolescents. Assessment and inclusion were executed face-to-face. Due to organizational reasons, the assignment of treatment methods could not be randomized. Adherence was investigated by measuring the number of completed modules. Outcome measures were collected by self-assessment questionnaires including the Revised Children’s Anxiety and Depression Scale and Avoidance and Fusion Questionnaire for Youth, as well as interviews using the Children’s Global Assessment Scale. The analysis was performed as an exploratory evaluation using descriptive data for treatment adherence and nonparametric within-group analysis with the Wilcoxon signed rank test for related samples and treatment outcomes. This evaluation is naturalistic, and the results are preliminary and of a hypothesis-generating character and should be handled with caution.

**Results:**

The iACT group without parental support (n=9) exhibited a gradual dropout throughout the treatment period (n=5), whereas the iACT group with parental support (n=15) exhibited the lowest number of dropouts from treatment before completion (n=2), of which all occurred during the second half of treatment. The within-group, per-protocol analyses for the Revised Children’s Anxiety and Depression Scale indicated reduced symptoms of anxiety and depression at the 12-month follow-up (*z* score: –2.94; *P*=.003; *r*=–0.6). The within-group, per-protocol analyses for the Avoidance and Fusion Questionnaire for Youth indicated increased psychological flexibility at the 12-month follow-up (*z* score: –2.54; *P*=.01; *r*=0.55). Nevertheless, no differences in overall functioning measured by the Children’s Global Assessment Scale were found.

**Conclusions:**

The results indicate that parental support might play a role in treatment adherence in iACT for adolescents with anxiety. Moreover, the outcome measures suggest that iACT for adolescents in primary care could constitute an effective treatment for both anxiety and depression, as indicated by the symptom reduction and increased psychological flexibility, maintained at the 12-month follow-up. Nevertheless, due to a small and gender-biased sample size with a large proportion of dropouts and missing data, a nonrandomized assignment of intervention, and an analysis limited to within group, this study should be considered an explorative evaluation rather than an outcome study.

## Introduction

According to the National Board of Health and Welfare in Sweden, mental health among children and young people has deteriorated, both based on self-reported psychological symptoms and on diagnosed mental disorders [1]. Anxiety and depression have been identified as significant factors, of which anxiety is the most common [2]. Furthermore, both anxiety and depression are associated with social withdrawal, adverse effects on academic performance, functional impairment, and ultimately, risk factors for suicide [3,4]. For adolescents with generalized anxiety disorder (GAD), separation anxiety disorder, and social phobia, the selective serotonin reuptake inhibitor Sertraline, as well as cognitive behavioral therapy (CBT), are the recommended treatment methods [1]. According to the Swedish National Board of Health and Welfare, CBT is well documented, recommended as a first intervention [5], and considered an effective treatment for anxiety disorders in children and adolescents [6].

In Sweden, primary care services include performing an initial assessment on children and adolescents regarding symptoms, symptom severity, and eventual need for treatment. If an anxiety disorder is assessed to be mild, an intervention shall be offered, and if moderate to severe, the patient shall be triaged to psychiatric care [5]. Furthermore, early intervention is crucial in preventing chronic mental illness [7], but many adolescents do not seek help from mental health care. O’Dea et al [8] have identified a lack of awareness among the population of signs of mental ill health, limited access to health care, and costs as possible obstacles. The authors suggest internet-based CBT (iCBT) as a way of increasing accessibility to treatment while reducing costs and that iCBT exhibits similar effect sizes as face-to-face treatment.

In a systematic review and meta-analysis, Vigerland et al [9] evaluated 25 studies on iCBT, including only studies in which the mean participant age was younger than 18 years. Of the studies, 7 studies were based on a Swedish population and 6 studies were on anxiety. The authors concluded that iCBT has positive outcomes, may be feasible, and exhibited moderate effect sizes compared to the waitlist. In a Danish randomized controlled trial (RCT), 70 adolescents with anxiety disorders were randomized into either iCBT or waitlist, and the iCBT group exhibited significant improvement based on both adolescent and parent ratings and that iCBT exhibited moderate to large effect sizes between groups [10]. Moreover, in an Australian RCT, 115 adolescents with anxiety from a community sample were randomized to either iCBT, face-to-face CBT, or waitlist conditions. At the 12-month follow-up, the authors found no significant differences regarding treatment outcomes between the groups and concluded that iCBT offers reduced therapist time and hence increased accessibility [11]. In a Swedish study, 120 adolescents were randomized to either standard iCBT, iCBT with learning support, iCBT with chat, or iCBT with learning support and chat. The group with learning support initially exhibited better outcomes but the difference was not sustained at the 6-month follow-up, and the authors determined iCBT to be an effective treatment method for adolescents with anxiety and depression. The authors found small effect sized on secondary outcomes related to anxiety and that the effect sized indicated the benefits of memory support during iCBT [12]. Nevertheless, in all the abovementioned studies, the participants were recruited either via advertisements or referral to secondary care. Thus, none of the studies were conducted in regular primary care.

Acceptance and commitment therapy (ACT) is a third-wave behavioral therapy oriented at acceptance and mindfulness and aimed at increasing psychological flexibility, defined as the ability to be present and act accordingly in line with one’s values [13]. Face-to-face ACT is considered an effective treatment for children and adolescents with anxiety disorders [14,15] and exhibits small to medium effect sizes regarding anxiety and depression [16]. In a Swedish study, Nissling et al [17] investigated the effectiveness of internet-delivered ACT (iACT) on adolescents with anxiety by randomizing 52 participants aged 15-19 years from all over Sweden into either iACT or waitlist. Both groups improved but the participants in the intervention group exhibited significantly higher improvements regarding anxiety and exhibited moderate effect sizes between groups. The authors concluded that iACT is effective in improving quality of life and psychological flexibility, which in turn was associated with reduced anxiety symptoms. Another study randomized 348 adolescents to either (1) iACT student coach and a digital coach group, (2) only iACT digital coach group, or (3) no intervention. The authors found significant improvements in the iACT groups compared to the control group regarding reduced anxiety and increased valued action and self-compassion [18].

Few studies have examined iACT for adolescents in a routine primary care setting. To broaden the understanding of iACT in primary care for adolescents with anxiety, the Internet Mediated Psychological Treatment-Acceptance and Commitment Therapy (IMPACT) project was conducted as an ongoing evaluation during the implementation of iACT for adolescents in the region Västra Götaland in southwestern Sweden. The intervention in focus is the same as that in the study by Nissling et al [17] and contains ACT features [19].

In the first IMPACT paper, the authors highlighted the importance of parental involvement in iACT for adolescents, suggesting it might compensate for low treatment motivation [20]. In the second IMPACT paper, the authors concluded that the role of the parents needs clarification [21]. Attention to parental engagement in mental health treatments of adolescents has increased in recent years, and in 2015, a review of 23 papers was conducted by Haine-Schlagel and Walsh [22]. The results indicated potential links between parental participation and positive outcomes. The authors concluded that further research is needed to determine treatment factors, as well as organizational factors, regarding parental engagement in mental health treatment for both children and families. Moreover, Lundkvist-Houndoumadi et al [23] performed a phenomenological analysis of 24 semistructured interviews with Danish families in which the youth received CBT for anxiety with parental involvement. The authors concluded that the therapists’ expectations of the parents to be cotherapists were difficult to implement in some cases due to the family dynamics and the expectations and resources among the parents. Overall, there seems to be a need for further information regarding the parental role in iCBT and iACT for adolescents and how the parents can support the adolescent’s treatment. Haine-Schagel and Walsh [22] have concluded that research regarding parental engagement would benefit from more studies on specific parent-supportive behaviors in clinical interactions [22].

In summary, few studies have investigated iACT for adolescents in a routine primary care setting nor the role of the parents. Therefore, the IMPACT project aimed to conduct an ongoing evaluation of introducing iACT for adolescents with anxiety in primary care. This is the third part of the IMPACT project and is aimed at conducting a follow-up 12 months after receiving iACT with or without parental support. The primary outcomes consist of treatment adherence and symptoms of anxiety and depression, and the secondary outcomes consist of psychological flexibility and overall functioning in adolescents. Thus, the aims of this evaluation can be concretized as follows:

1. Is there a connection between parental support and adherence to iACT for adolescents with anxiety?
2. Does iACT for adolescents with anxiety result in decreased symptoms of anxiety and depression between pretreatment and 12 months after terminating treatment?
3. Does iACT for adolescents with anxiety result in increased psychological flexibility between pretreatment and 12 months after terminating treatment?
4. Does iACT for adolescents with anxiety result in improved overall functioning between pretreatment and 12 months after terminating treatment?

## Methods

In this section, the study design, participants, procedure, intervention, measures, data analysis, and ethical considerations are presented.

### Study Design

Initially, the intention was to perform a follow-up of iACT during the implementation phase in primary care and to conduct between-group analyses. However, due to organizational limitations, the authors instead opted for a pragmatic approach to the data. Consequently, the analysis was converted into an exploratory evaluation of iACT for adolescents with anxiety in primary care.

The IMPACT project was conducted within the regular patient flow during the implementation phase and due to organizational reasons, randomization of the participants could not be made. Using a non-RCT, the therapists assigned the participants to either iACT with or without parental support or treatment as usual (TAU), consisting of face-to-face treatment for anxiety individually or in a group format. Therefore, the authors had limited insight into the assignment process. Therefore, this study is naturalistic and the results are preliminary and of a hypothesis-generating character.

Quantitative data were collected before, during, and after treatment, and follow-ups were performed 6 and 12 months after terminating treatment. In this evaluation, pretreatment and the 12-month follow-up are being compared. Due to difficulties in recruiting therapists, the sample size is relatively small, which further decreases the quality of the data, furthermore, the amount of missing data is relatively large.

No a priori power analysis was conducted, so between-group analyses could not be made. Therefore, the TAU group is not included in this evaluation, adherence measures are analyzed using descriptive data, and outcome measures are analyzed using within-group analyses. Therefore, the results are treated as an explorative evaluation of iACT in practice rather than a scientific study.

### Participants

The participants were recruited from adolescents seeking help in primary care for anxiety symptoms at 3 different health care centers located in southwestern Sweden and specialized in treating adolescents with mental health issues. Previously, there was no iACT program for young people in Sweden, and the treatment program was developed and adapted for the 13-18 years age group, hence the age group that was studied. The inclusion criteria consisted of being aged 13-18 years; having access to a computer, iPad, or smartphone with internet access; being able to read and write in Swedish; and having been diagnosed with mild to moderate anxiety such as GAD, social phobia, panic disorder, or unspecified anxiety disorder. The exclusion criteria consisted of having a neuropsychiatric diagnosis, intellectual disability, bipolar disease, suicidality, or ongoing psychotherapeutic treatment or daily consumption of benzodiazepines.

This evaluation originally included 35 participants aged 13-18 years. Of these participants, 9 participants received iACT without parental support; 15 participants received iACT with parental support; and 11 participants received TAU, of which, 8 participants received group therapy and 3 participants received individual therapy. Besides providing iACT, 2 of the health care centers involved in the study only provided group therapy, whereas the third only provided individual therapy. Since no power analysis was performed before the data collection, comparisons between groups could not be made, hence the TAU group was excluded from this evaluation. Table 1 demonstrates the distribution of age and gender among the participants.

**Table 1:** The distribution of age and gender among the participants.

Variable			Frequencies			
			iACT^a^ without parental support (n=9)	iACT with parental support (n=15)		TAU^b^ (n=11)
**Age group (years), n (%)**						
	13-15	8 (89)		10 (67)	9 (82)	
	16-18	1 (11)		5 (33)	2 (18)	
**Sex, n (%)**						
	Female	8 (89)		15 (100)	10 (91)	
	Male	1 (11)		0 (0)	1 (9)	
	Other	0 (0)		0 (0)	0 (0)	

^a^iACT: internet-delivered acceptance and commitment therapy.

^b^TAU: treatment as usual.

### Procedure

Patients aged 13-18 years, accompanied by a parent, seeking help in primary care for anxiety problems were informed about the study and were offered participation by the therapist. All the adolescents included provided verbal consent and the parents provided written consent prior to participation. The patient and the parent participated in an assessment and inclusion meeting conducted by a participating therapist. The parent was subsequently led to another room to fill in a questionnaire whereas the adolescent was interviewed further.

After the assessment, all the participants who met the inclusion criteria were assigned by the therapist to either iACT, with or without parental support, or TAU. The assignment of groups was not randomized, and the authors have no information on how many patients were excluded from the study by the therapists nor how the therapists assigned the patients into groups.

Furthermore, the participating adolescents completed questionnaires before, during, and after treatment, as well as 6 and 12 months after treatment termination, and participated in diagnostic clinical interviews before and after treatment, as well as 12 months after treatment. To ensure the integrity of the adolescents and data security, the forms were distributed via the survey platform Esmaker [24], if possible, and otherwise in paper format. The paper forms and interview protocols were also added to a research journal to collect additional data such as other ongoing treatments.

The recruitment process took place from 2018 to 2020, hence parts of the data collection coincided with the COVID-19 pandemic, during which some upper secondary schools in Sweden introduced distance learning for periods of time while other schools did not [25]. It is possible that the pandemic affected the number of participants in the study. Furthermore, due to the difficulty of recruiting therapists, the number of participants in the study is relatively low. Despite the low number of participants, the recruitment was terminated due to financial reasons.

### Intervention

In this study, the participants were recruited from 3 health care centers that were specialized in adolescent mental health and located in southwestern Sweden: Gothenburg, Borås, and Uddevalla. These centers form a part of primary care and are specialized in helping patients aged 6-18 years. The therapists in this study were either licensed psychologists or intern psychologists, and as a part of implementing iACT at these centers, they participated in a 2-day course and received specific training in iACT for adolescents.

### iACT Without Parental Support

These participants received a guided, internet-based, self-help program called Anxiety Help for Adolescents (in Swedish: Ångesthjälpen Ung) developed by Psykologpartners W&W AB. The program is adapted for patients aged 13-19 years with mild to moderate anxiety, for example, social phobia, GAD, panic disorder, obsessive-compulsive disorder, or unspecified anxiety disorder [19].

The iACT intervention consists of 8 modules and the recommended treatment duration is 10 weeks with weekly feedback from the therapist. The program is adapted to the target group of adolescents regarding formulations, concretizations of theoretical concepts, and clinical examples, as well as the overall structure, and presents different strategies through text, videos, exercises, and forms. There is a messaging function in which the therapist and the patient can communicate asynchronously, and the therapist can initiate conversations through telephone, video calls, or physical meetings at the clinic. The therapist supports the patient through motivation, giving feedback, answering questions, and prompting upcoming parts of the program [19]. For a detailed list of contents of the iACT intervention, see Multimedia Appendix 1.

### iACT With Parental Support

These participants were assigned to the iACT program described above, with the addition of receiving parental support on how to support their adolescent’s anxiety regulation. Both the participants and parents were initially given information about the content and structure of the iACT program [19]. Subsequently, the parents took part in 3 physical meetings during their adolescent’s treatment period, either individually or in groups, and were conducted with the help of a manual (Multimedia Appendix 2)*.* The content included psychoeducation about anxiety and different reactions, examples of different anxiety disorders, and behavioral strategies to handle anxiety such as exposure, relaxation, breathing, balance activity, and rest. All the information was condensed into a pamphlet called More Than Afraid (in Swedish: Mer än rädd) [26].

### TAU Group

The participants in the TAU group received the treatment they would normally be offered at the clinics, consisting of face-to-face treatment for anxiety individually or in a group format, both 8 weeks long, as conventional in clinical settings. The TAU group is not included in this evaluation since the groups could not be compared.

### Measures

The adolescent filled in the following forms before, during, and after treatment, as well as 6 and 12 months after treatment:

- Revised Children’s Anxiety and Depression Scale (RCADS-Children, 47 items), consisting of the 2 main scales, anxiety and depression, and the 6 subscales, social phobia, panic disorder, GAD, compulsive disorder, separation anxiety, and depression, on which higher scores indicate a higher number of symptoms. The subscales exhibit a high internal consistency (α=.78-.88) in a sample of 513 children in the United States [27,28].
- Avoidance and Fusion Questionnaire Youth (AFQ-Y8), designed to measure the level of psychological flexibility in youth aged 12-20 years: higher scores indicate higher levels of psychological inflexibility. In a Swedish sample of 62 children undergoing cancer treatment, AFQ-Y8 exhibited acceptable internal consistency (α=.76), good test-retest reliability (ICC=0.64), and convergent validity (*r*=0.42) [29].

Furthermore, the adolescent was interviewed before and after treatment and 12 months after completion. The assessments were performed by a psychologist or intern psychologist using the following measures:

- The Mini International Neuropsychiatric Interview for Children and Adolescents was used for diagnostics [30] and exhibits validity and test-retest reliability comparable to other standardized screening tools [31,32].
- The Children’s Global Assessment Scale (CGAS) was used to assess overall functioning. The interviewer performs an assessment of the adolescent’s level of functioning on a scale from 1 to 100, of which a higher score indicates a higher level of functioning [33]. CGAS exhibits high interrater and test-retest reliability, as well as high discriminant and concurrent validity [34-36].

### Data Analysis

This study aimed to investigate whether there is a connection between parental support and adherence to iACT for adolescents with anxiety and whether the treatment results in differences in symptoms of anxiety and depression, psychological flexibility, and overall functioning at 12 months after terminating treatment.

Due to a nonrandomized design, a small sample, a large dropout, and the fact that no a priori power analysis was made, the data are nonparametric, which makes between-group comparisons less meaningful. Therefore, the TAU group (n=11) is not included in this evaluation. Adherence was analyzed using descriptive data and Meier-Kaplan survival analysis, a statistical method used for measuring the distribution of time of occurrences in cohort groups [37]. In this study, dropout is defined as terminating the iACT program before the last module. Participants with missing time-to-event data were excluded from the Kaplan-Meier analysis. Meanwhile, the outcome measures were analyzed using within-group comparisons.

Adherence was analyzed for all participants receiving iACT without parental support (n=9) and with parental support (n=15), presented in separate groups. In contrast, for the outcome measures, all participants receiving iACT are presented as 1 group, including both with and without parental support due to small groups. In the outcome measures, the pretreatment measurement and the 12-month follow-up were compared and only included participants completing both the premeasurement and the 12-month follow-up.

For the outcome measures, patient-rated scores using RCADS and AFQ-Y8, as well as therapist-rated scores using CGAS and within-group analyses were performed using the nonparametric statistical method Wilcoxon signed rank test for related samples. Effect sizes were calculated based on the formula described by Field [38] and were interpreted as 0.10-<0.3 (small effect), 0.30-<0.5 (moderate effect), and ≥0.5 (large effect). The within-group analyses were performed in SPSS Statistics (version 29; IBM Corp).

### Ethical Considerations

This evaluation constitutes a part of the research project IMPACT in 2017-2021 (Swedish National Research Register; ID: 240221), approved by the Regional Ethics Committee in Gothenburg (Dnr: 703-17). The IMPACT project was designed to conduct an ongoing evaluation of introducing iACT for adolescents with anxiety in primary care during the implementation phase, and this evaluation is a 12-month follow-up. The participants have been informed that their participation is voluntary and that they have the right to cancel without further explanation. Moreover, the participants have been informed that participation in the study will not in any way affect their future opportunities for care and treatment at the health center and that participation in the study will not be mentioned in medical records. Both accessing care and participating in the study were free. Furthermore, the participants have been informed about how the data will be managed, including confidentiality aspects, as well as analysis and presentation. The confidentiality of all participants is thus guaranteed, and consent from all participants has been obtained including both adolescents and parents. Digital forms were collected using the survey platform Esmaker [24], and data were analyzed using SPSS Statistics.

## Results

### Overview

**Figure 1:**
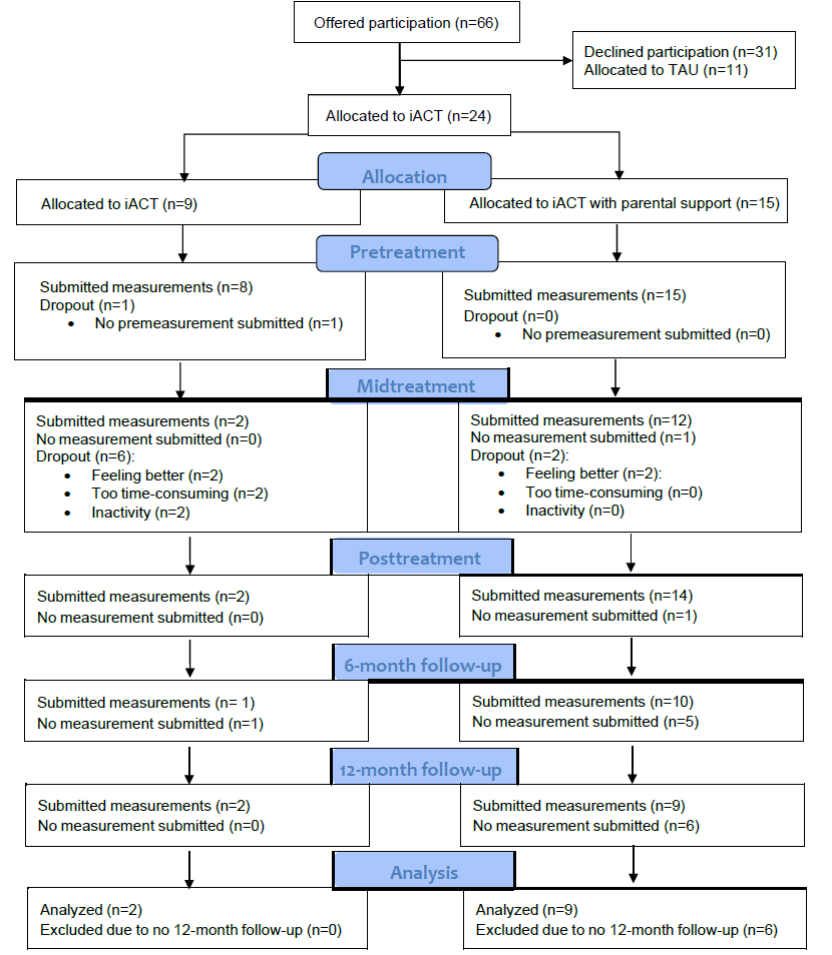
Consolidated Standards of Reporting Trials (CONSORT) Flow diagram of internet-delivered Acceptance and Commitment Therapy (iACT) and treatment as usual (TAU).

In this section, the results are presented regarding treatment adherence and outcome measures based on the questions of the aims. Adherence is presented for all participants (n=35), and the participants receiving iACT are presented in 2 separate groups: iACT without parental support and iACT with parental support. The outcome measures are presented for the participants who completed the assessments for both the pretreatment and the 12-month follow-up (n=11). Moreover, the iACT participants are presented as 1 group, regardless of whether they have received parental support or not. Below, the primary outcomes are presented. Figure 1 demonstrates a CONSORT (Consolidated Standards of Reporting Trials) flow diagram of the study.

### Is There a Connection Between Parental Support and Adherence to iACT for Adolescents With Anxiety?

For the analyses regarding adherence to treatment, the participants were presented in 2 groups: iACT without parental support (n=9) and iACT with parental support (n=15). Table 2 demonstrates descriptive statistics for the number of completed modules or sessions at the time of terminating the program.

**Table 2:** Descriptive statistics for the number of completed modules.

Completed modules or sessions(%)	iACT^a^ without parental support (n=9), n (%)	iACT with parental support (n=15), n (%)
<25	1 (11)	0 (0)
25-50	2 (22)	0 (0)
50-75	1 (11)	1 (7)
75-100	3 (33)	12 (80)
Missing	2 (22)	2 (13)

^a^iACT: internet-delivered acceptance and commitment therapy.

The participants receiving iACT without parental support (n=9) exhibited a gradual dropout rate throughout treatment, of which 5 participants dropped out before treatment completion. In contrast, the participants receiving iACT with parental support (n=15) exhibited the least number of dropouts (n=2), of which all occurred during the second half of the treatment.

**Figure 2:**
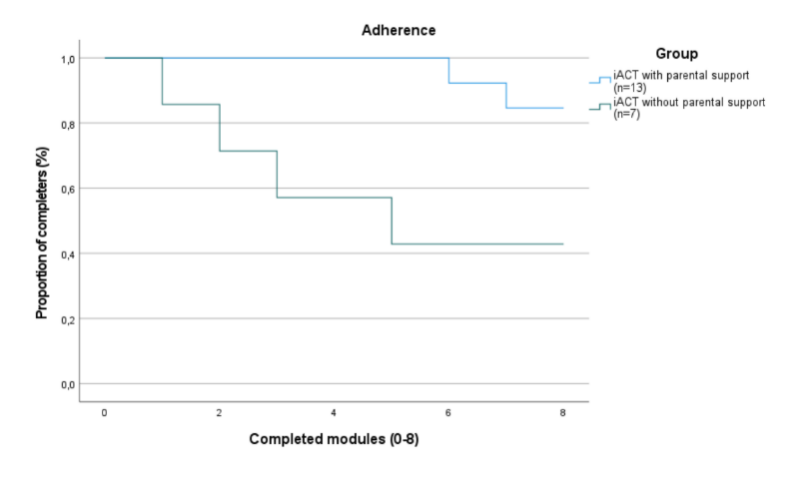
Proportion of dropouts between groups of internet-delivered Acceptance and Commitment Therapy (iACT) with or without parental support.

Figure 2 demonstrates a Meier-Kaplan graph illustrating the dropouts. The x-axis represents at which module the dropout occurred, with 0 indicating dropout before initiating treatment and 8 representing complete treatment. The y-axis represents the percentage of participants remaining in treatment.

### Does iACT for Adolescents With Anxiety Result in Decreased Symptoms of Anxiety and Depression Between Pretreatment and 12 Months After Terminating Treatment?

In this section, the results of the patient-rated RCADS scores are analyzed for all participants completing both the premeasurement and the 12-month follow-up (n=11). In this section, the participants receiving iACT are in the same group, regardless of whether they have received parental support or not. Table 3 demonstrates descriptive statistics for the therapist-rated measurement points on RCADS.

**Table 3:** Descriptive statistics for the measurement points on RCADS^a^.

iACT^b^ (n=11)			Score, mean (SD)		Score, median (IQR)		Score, range
**RCADS**							
	Pretreatment	71.7 (17.5)		70 (65-86)		41-104	
	Posttreatment	52.9 (22.3)		52 (38-71)		25-98	
	6-month follow-up	47.1 (26.9)		40 (27-63)		13-104	
	12-month follow-up	49.3 (25.4)		44 (32-63)		18-111	
**RCADS—Anxiety**							
	Pretreatment	58.6 (14.1)		58 (52-71)		33-84	
	Posttreatment	42.9 (17.9)		42 (32-53)		21-83	
	6-month follow-up	38.5 (22.4)		30 (22-46)		12-87	
	12-month follow-up	39.4 (19.8)		39 (24-49)		15-88	
**RCADS—Depression**							
	Pretreatment	13.1 (4.4)		14 (8-17)		6-20	
	Posttreatment	10.0 (5.4)		10 (5-16)		3-18	
	6-month follow-up	8.5 (5.3)		9 (4-12)		1-17	
	12-month follow-up	9.8 (6.2)		8 (5-14)		3-23	

^a^RCADS: Revised Children’s Anxiety and Depression Scale.

^b^iACT: internet-delivered acceptance and commitment therapy.

The results from the Wilcoxon signed rank test for related samples for the RCADS total scores demonstrated a decrease in symptoms of anxiety and depression and a large effect size for the RCADS total scores from preassessment to the 12-month follow-up (*z* score: –2.81; *P*=.005; *r*=0.60). When analyzing anxiety and depression scores separately by subscales, reductions between the pretreatment assessment to the 12-month follow-up assessment for both anxiety (*z* score: –2.81; *P*=.005; *r*=0.60) and depression (*z* score: –2.67; *P*=.008; *r*=0.57) and large effect sizes were obtained. Below, the secondary outcomes are presented.

### Does iACT for Adolescents With Anxiety Result in Increased Psychological Flexibility Between Pretreatment and 12 Months After Terminating Treatment?

In this section, the results of the patient-rated AFQ-Y8 scores are analyzed, indicating the adolescent’s self-rated levels of psychological flexibility. In this section, the participants receiving iACT are presented in the same group, regardless of whether they have received parental support or not. Table 4 demonstrates descriptive statistics for the therapist-rated measurement points on AFQ-Y8 and CGAS.

**Table 4:** Descriptive statistics for the measurement points on AFQ-Y8^a^ and CGAS^b^.

iACT^c^ (n=11)			Score, mean (SD)		Score, median (IQR)		Score, range
**AFQ-Y8**							
	Pretreatment	18.8 (5.8)		21 (12-22)		11-30	
	Posttreatment	14.9 (7.7)		13 (10-20)		3-30	
	6-month follow-up	12.3 (7.6)		11 (7-17)		3-27	
	12-month follow-up	12.7 (6.7)		11 (8-17)		5-29	
**CGAS**							
	Pretreatment	64.1 (5.8)		65 (60-65)		55-75	
	Posttreatment	72.7 (13.3)		75 (55-85)		55-90	
	12-month follow-up	70.0 (14.3)		70 (55-85)		45-85	

^a^AFQ-Y8: Avoidance and Fusion Questionnaire for Youth.

^b^CGAS: Children’s Global Assessment Scale.

^c^iACT: internet-delivered acceptance and commitment therapy.

The results from the Wilcoxon signed rank test for related samples for the AFQ-Y8 demonstrated increased psychological flexibility from preassessment to the 12-month follow-up (*z* score: –2.54; *P*=.01; *r*=0.55).

### Does iACT for Adolescents With Anxiety Result in Improved Overall Functioning Between Pretreatment and 12 Months After Terminating Treatment?

In this section, the results of the therapist-rated CGAS scores are presented. The results from the Wilcoxon signed rank test on the CGAS total scores indicated no difference between measurement points from pretreatment to the 12-month follow-up for the iACT group (*z* score: –0.51; *P*=.96; *r*=0.146).

## Discussion

### Overview

This evaluation aimed to investigate whether there is a connection between parental support and adherence to iACT for adolescents with anxiety and whether iACT for adolescents with anxiety results in a difference in symptoms of anxiety and depression, psychological flexibility, and overall functioning, between the pretreatment measurement and 12 months after terminating treatment. In this section, the principal findings, limitations, and implications will be discussed in contrast to other studies. Overall, the results must be handled with caution due to the nonrandomized design, small sample size, and large amount of missing data.

### Principal Findings

#### Overview

The IMPACT project was conducted as an ongoing evaluation to broaden the understanding of iACT for adolescents with anxiety in a routine primary care setting during the implementation phase. In the first IMPACT paper, Weineland et al [20] concluded that the interviewed therapists were positive to iACT for adolescents but also identified challenges such as motivating patients. In the second paper, Lilja et al [21] found that the interviewed parents expressed uncertainty about their role in the treatment and clearer parental treatment support was suggested. This is the third part of the IMPACT project, consisting of a follow-up on adolescents with anxiety 12 months after receiving iACT, with or without parental support regarding treatment adherence, symptoms of anxiety and depression, and psychological flexibility, as well as overall functioning. The primary outcomes are discussed below.

#### Is There a Connection Between Parental Support and Adherence to iACT for Adolescents With Anxiety?

Regarding treatment adherence, the participants receiving iACT with parental support exhibited later and fewer dropouts than the participants receiving iACT without parental support. These findings might be due to the idea suggested by Weineland et al [20] that parental support could compensate for low treatment motivation among adolescents. However, due to the nonrandomized design and small sample size in this, further research is needed to test this hypothesis in RCTs and with larger samples. Other potential mediating effects could be giving the parents a deeper understanding of anxiety in both themselves and the adolescent, as well as how to support their adolescent and function as a cotherapist alongside the therapist. In addition, in each group, 2 participants discontinued treatment due to feeling better, indicating that dropouts from treatment are not necessarily negative.

In previous research, the authors have pointed to potential connections between parental support and positive treatment outcomes in children and families [22], including that some parents need support in their role as cotherapists in treatment [23]. The current research on the role of motivation in iACT for adolescents with anxiety is currently limited. Nevertheless, in a Norwegian study, Fjermestad et al [39] concluded that motivation predicts early alliance in CBT for youth with anxiety. Furthermore, in a Danish study by Stjerneklar et al [10], both the parents and the therapists were encouraged to help motivate the adolescent in their iCBT, in which iCBT exhibited moderate to large effect sizes between groups on anxiety compared to the waitlist.

Furthermore, iACT with parental support can be considered a complex intervention, which can be defined as an intervention consisting of multiple components. Complex interventions cause challenges in the development and identification [40] and Hasson and von Thiele Schwartz [41] claim that complex interventions tend to be at a disadvantage in research due to the difficulty in isolating them from the context. The authors argue that this applies to a large amount of psychological treatment methods, compared to medical treatments.

#### Does iACT for Adolescents With Anxiety Result in Decreased Symptoms of Anxiety and Depression Between Pretreatment and 12 Months After Terminating Treatment?

Outcome measures were investigated using a within-group analysis, in which the iACT-group demonstrated reduced symptoms of anxiety and depression between the preassessment and 12-month follow-up. Multiple previous studies have indicated positive treatment outcomes for iACT for adolescents with anxiety [9-12], but few have performed follow-ups at 12 months after treatment or longer. In the meta-analysis by Vigerland et al [9], of the papers on iACT for children and adolescents with anxiety, 2 papers had a 1-year follow-up: Spence et al [11] concluded that the improvements in both CBT and iACT were maintained at the 12-month follow-up and Tillfors et al [42] discovered significant improvements in iACT for high school students with anxiety disorder, maintained at the 12-month follow-up. Nevertheless, none of the studies included follow-ups more than 1 year after treatment, hence the long-term effects of iACT for adolescents with anxiety should be investigated further.

In this evaluation, in 2 of the cases, the results of Mini International Neuropsychiatric Interview for Children and Adolescents and CGAS exhibited an increase in symptoms and a decrease in functioning between the post and 12-month follow-up. Therefore, the interviewer had the impression that the COVID-19 pandemic influenced the results. In both cases, of which 1 participant from the iACT group and 1 from the TAU group, the participant was diagnosed with social phobia at the 12-month follow-up and described that at least some of the symptoms were due to returning from distance to classroom learning. In addition, the authors suspect that the pandemic itself could have affected the anxiety levels and functioning of the participants, for example, fear of the disease itself or uncertainty about the future.

In a Swedish study, the authors surveyed 1818 adolescents, of which approximately 80% transferred to distance learning during the pandemic. The authors concluded that most of the participants experienced decreased mental health, especially female participants and those in distance learning. The authors also discovered that distance learning could result in less victimization and poorer mental health overall [43]. In another Swedish study, 3068 participants aged 16-17 years filled in a questionnaire about the impact of the COVID-19 pandemic from December 2020 to March 2021. The author concluded that female participants reported more worry than male participants and that participants with a lower socioeconomic background reported higher levels of worry in general, except for climate anxiety [44].

In an international systematic review and meta-analysis of 74 papers on anxiety among children and adolescents during the pandemic, the authors concluded that anxiety levels were more prevalent among female participants than male participants in North America and Europe than South America and Asia, during the second wave of COVID-19 and school closures [45]. A Finnish study surveyed 450,000 participants aged 13-20 years about the pandemic. The authors discovered that social anxiety increased from 2013 to 2021, especially among the female participants, and that unmet needs for schoolwork support, and fear of getting infected by COVID-19 or transmitting it to others were associated with high levels of social anxiety. Nevertheless, the authors observed no clear connection between time spent in distance learning and levels of social anxiety [46].

Furthermore, in an American study, 280 high schoolers were surveyed on social anxiety and the use of technology. The author discovered a positive relationship between social anxiety and a preference for using technological communication instead of face-to-face communication [47]. In an international study, 2665 participants aged 18-25 years from 121 countries, of which the majority from Australia, the United States, and the United Kingdom, were surveyed on social restrictions related to COVID-19 and its effect on loneliness, social anxiety, and depression. The authors concluded that reductions in social restrictions resulted in an increase in social anxiety due to having to readjust to the social environment [48]. In other words, the relationship between the COVID-19 pandemic and anxiety among youth is a complex matter with a diversity of outcomes, of which multiple possible scenarios might have affected the results of this study.

In this study, COVID-19 can be considered a confounding factor, which Jager et al [49] defined as a risk factor, unequally distributed among the participants, and not included in the causal pathway. Pourhoseingholi et al [50] define confounding factors as a variable affecting the variables studied but not their relationship. To prevent or reduce the confounding factors, Jager et al [49] suggest using exclusion criteria, for example, participant age, randomizing the assignment to groups, matching participants for example in pairs with or without exposure, or stratifying the participants into subgroups. Pourhoseingholi et al [50] mention that stratification is suitable with a low number of strata, whereas multivariate models, such as analysis of covariance, as well as logistic and linear regression, can be used with a larger number of covariates and confounders. The secondary outcomes are discussed below.

#### Does iACT for Adolescents With Anxiety Result in Increased Psychological Flexibility Between Pretreatment and 12 Months After Terminating Treatment?

In this evaluation, the analyses of AFQ-Y8 demonstrated an increase in psychological flexibility between the pretreatment measurement and the 12-month follow-up. These results are in line with previous research, concluding iACT to be effective in reducing symptoms of anxiety and increasing psychological flexibility, as well as suggesting a possible link between anxiety levels and psychological flexibility [17].

#### Does iACT for Adolescents With Anxiety Result in Improved Overall Functioning Between Pretreatment and 12 Months After Terminating Treatment?

In this evaluation, the analyses of overall functioning using CGAS did not indicate differences between the pretreatment measurement and the 12-month follow-up. However, in a meta-analysis of 9 RCTs on iACT for pediatric anxiety disorders, of which 7 included CGAS as a measure of functioning, the authors concluded that their confidence in the effect of iACT on functioning is low [51].

In summary, the results of this evaluation support a possible connection between parental support and adherence to iACT for adolescents with anxiety. Further research is needed to investigate the nature of the connection. Furthermore, the analyses of the outcome measures indicate reduced symptoms of anxiety and depression and increased psychological flexibility between the pretreatment measurement and the 12-month follow-up but no difference regarding overall functioning. However, due to a nonrandomized design, a small sample size, and a large amount of missing data, the results are uncertain, and the generalizability is severely limited.

### Limitations

In this evaluation, the participants were recruited within the regular patient flow in primary care, which on one hand increases the ecological validity of the study but on the other hand decreases the control of several third variables influencing the groups. Performing pragmatic evaluations on how the treatment method works under regular conditions is a concrete way to achieve local evidence. Evaluating iACT the way it is provided in clinical practice, without adding resources or excluding patients for the sake of the evaluation, can provide a closer input on the actual effect, which in turn can increase the external validity.

Due to organizational problems in conducting the study, the participants could not be randomized and were instead assigned to iACT or TAU following a nonrandomized design. Since the authors could not control the assignment of treatment method, no conclusions on eventual differences in treatment outcomes between groups could be made. Therefore, the TAU group was omitted from this evaluation and the internal validity is reduced.

This evaluation took place during the implementation phase of iACT in primary care, which poses its own unique challenges. Hasson and von Thiele Schwarz [41] suggest that performing follow-ups and giving feedback are ways to increase the motivation to implement a new method. In other words, the evaluation itself can influence the object of evaluation. During the IMPACT project, one of the staff members at one of the clinics involved mentioned that the project helped them initiate the implementation.

For the therapists involved in this study, the implementation phase included a learning phase in assessing and assigning iACT to patients. In the first IMPACT paper, the interviewed therapists discussed which patients iACT can be helpful for. The therapists concluded that iACT is more suitable for patients with self-discipline, acceptance of personal responsibility, capability of introspection, and appreciation of working independently with the program. Furthermore, the therapists concluded that iACT is better suited for patients with anxiety rather than depression and that the symptoms should not be too severe, wide-ranging, or long-standing, and that the patient preferably should be in the upper teens. Furthermore, several therapists expressed that iACT is less suitable for patients with learning disabilities, neuropsychiatric illness, or dyslexia [20].

The participant recruitment took place between 2018 and 2020, hence parts of the data collection took place during the COVID-19 pandemic. The authors suspect that the pandemic influenced the anxiety and functioning of the participants both directly, such as fear of infection, and indirectly such as transferring between distance and classroom learning in some cases. Ideally, the authors could have addressed this during the study, for example, by specifically asking about the effect of the pandemic. Thus, the results should be handled with caution.

The sample size in this study is relatively small, especially in the subgroup analyses, which severely limits the generalizability of the results. Furthermore, this evaluation contained a relatively large amount of missing data in proportion to the sample size. In a review paper, Kang [52] reviews techniques for managing missing data and argues that the best method is prevention, for example, by minimizing the number of follow-ups. The data collection for the IMPACT project consisted of surveys before, during, and after treatment and at 6- and 12-month follow-ups, as well as interviews before, during, after, and 12 months after treatment. It is possible that the number of measurements might have had an impact on the participation, for example, by reducing the motivation to participate. On the other hand, a larger number of measurement points, as well as both written and spoken, results in more opportunities to collect data.

In this evaluation, missing data were handled by complete case analysis, which Kang [52] does not recommend with small sample sizes. Applying the last-observation-carried-forward method would have increased the amount of data included in the analyses. However, since the previous measurements occur closer to the treatment phase, this could also create a bias in the data. Furthermore, due to a large number of missing data, the authors determined that the last-observation-carried-forward method would risk being too misleading; therefore, only participants with complete data were included in the analysis.

Regarding dropouts, Kang [52] recommends documenting the reason and thus enabling further analysis. In this evaluation, treatment adherence was investigated by analyzing the timing and reason behind the dropouts. Nevertheless, more actions could have been taken in preventing and investigating the dropouts. In a Swedish RCT, 162 adults were investigated regarding their participation in iACT to explore variables predicting dropout, adherence, and outcomes. The authors concluded that the level of treatment credibility predicted dropouts whereas attrition was associated with higher levels of impulsivity and low levels of intrinsic treatment motivation [53].

The generalizability of the results in this evaluation is limited due to the small and gender-based sample of young people that were included. To achieve a better understanding of mental health in male adolescents and to enhance primary care services, it is important to address gender bias in future research and clinical work. Furthermore, the amount of missing data was relatively high, which further reduced the possibility of drawing conclusions based on the data.

Due to limitations in the study design regarding a small and gender-biased sample size with a large proportion of dropouts and missing data, a nonrandomized assignment of intervention, and analysis limited to within-group, this investigation should be considered an explorative evaluation of a new method rather than a scientific outcome study. Further research on iACT in the regular patient flow in primary care is needed.

### Conclusions

This evaluation consists of a follow-up on adolescents, 12 months after receiving iACT, with or without parental support. Due to a large amount of missing data, the results should be viewed as an evaluation rather than a scientific study. Adherence to treatment was investigated, indicating that parental support could increase adherence to iACT, which in turn might improve the conditions for young patients undertaking iACT treatment. The results also underscore the importance of parental involvement in the treatment of adolescents with mental illness. More research is needed to explore the relationship between parental support and treatment outcomes and how clinicians can facilitate the process.

Future research should investigate internet-based treatments for adolescents in primary care with additional, possibly digital, parental support programs in RCTs. After the IMPACT project was conducted, an internet-delivered parental support program was developed, in which information about how to support the adolescent during treatment was added [19]. Further research is needed on parental support in this format as well. Moreover, further research is needed on involving next of kin in health care in general.

Furthermore, the analyses of the outcome measures suggest that iACT might be an effective treatment for both anxiety and depression and has the potential to be an effective treatment of comorbidity and a broader spectrum of anxiety problems.

## Appendix

Multimedia Appendix 1 The contents of the internet-delivered acceptance and commitment therapy (iACT) intervention translated into English.

Multimedia Appendix 2 User manual for the Anxiety School (Användarmanual till Ångestskolan in Swedish).

## Abbreviations

ACT: acceptance and commitment therapy
AFQ-Y8: Avoidance and Fusion Questionnaire for Youth
CBT: cognitive behavioral therapy
CGAS: Children’s Global Assessment Scale
CONSORT: Consolidated Standards of Reporting Trials
GAD: generalized anxiety disorder
iACT: internet-delivered acceptance and commitment therapy
iCBT: internet-based cognitive behavioral therapy
IMPACT: Internet Mediated Psychological Treatment-Acceptance and Commitment Therapy
RCADS: Revised Children’s Anxiety and Depression Scale
RCT: randomized controlled trial
TAU: treatment as usual
