# Correction: The Impact of Parental Support on Adherence to Therapist-Assisted Internet-Delivered Acceptance and Commitment Therapy in Primary Care for Adolescents With Anxiety: Naturalistic 12-Month Follow-Up Study

**DOI:** 10.2196/82840

**Published:** 2025-10-29

**Authors:** Anna Larsson, Sandra Weineland, Linnea Nissling, Josefine L Lilja

**Affiliations:** 1 General Practice/Family Medicine, School of Public Health and Community Medicine, Institute of Medicine Sahlgrenska Academy University of Gothenburg Gothenburg Sweden; 2 Research, Education Development & Innovation, Primary Health Care Region Västra Götaland Borås Sweden; 3 Department of Psychology University of Gothenburg Gothenburg Sweden; 4 Department of Psychology Faculty of Health and Life Sciences Linnaeus University Växjö Sweden

In “The Impact of Parental Support on Adherence to Therapist-Assisted Internet-Delivered Acceptance and Commitment Therapy in Primary Care for Adolescents With Anxiety: Naturalistic 12-Month Follow-Up Study” (JMIR Pediatr Parent 2025;8:e59489), the data in Table 2 was incorrectly presented.

The corrected version of Table 2 is as follows:

**Table:** 

Completed modules or sessions (%)	(iACT) without parental support (n=9), n (%)	(iACT) with parental support (n=15), n (%)
<25	1 (11)	0 (0)
25-50	2 (22)	0 (0)
50-75	1 (11)	1 (7)
75-100	3 (33)	12 (80)
Missing	2 (22)	2 (13)

In the originally published article, the Methods section did not include information on how missing data were handled in the survival (Kaplan-Meier) analysis.

To address this, the following sentence has been added to the Methods section under “Data Analysis” on page 18:

“Participants with missing time-to-event data were excluded from the Kaplan-Meier analysis”.

The corrected version of that excerpt now reads:

Due to a nonrandomized design, a small sample, a large dropout, and the fact that no a priori power analysis was made, the data are nonparametric, which makes between-group comparisons less meaningful. Therefore, the TAU group (n=11) is not included in this evaluation. Adherence was analyzed using descriptive data and Meier-Kaplan survival analysis, a statistical method used for measuring the distribution of time of occurrences in cohort groups [37]. In this study, dropout is defined as terminating the iACT program before the last module. Participants with missing time-to-event data were excluded from the Kaplan-Meier analysis. Meanwhile, the outcome measures were analyzed using within-group comparisons.

Figure 2 has been updated to improve clarity and ensure accurate representation of the Kaplan-Meier survival analysis. The revised figure reflects the exclusion of participants with missing time-to-event data, aligning it with the updated Methods section. No changes were made to the underlying data or results.

**Figure 2:**
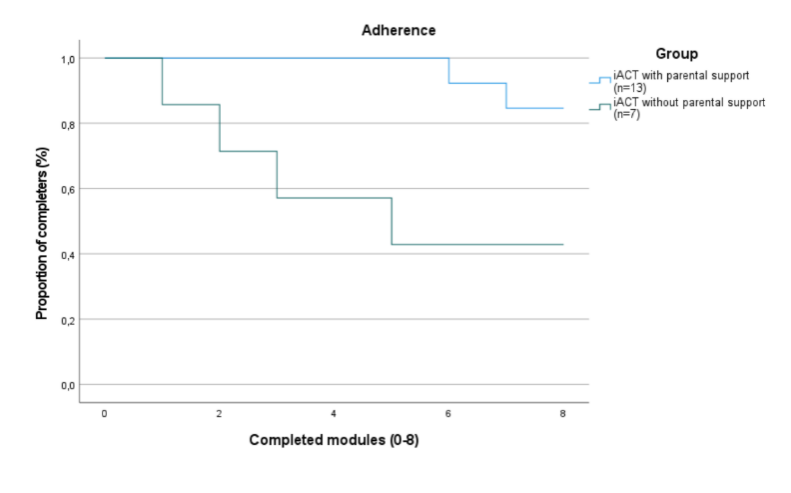
Proportion of dropouts between groups of internet-delivered Acceptance and Commitment Therapy (iACT) with or without parental support.

Finally, due to a change in affiliation after the original publication, the corresponding author's email address has been updated to:

psy.ac.larsson@gmail.com

The correction will appear in the online version of the paper on the JMIR Publications website, together with the publication of this correction notice. Because this was made after submission to full-text repositories, the corrected article has also been resubmitted to those repositories.

