# Supplementary material for: The Impact of Parental Support on Adherence to Therapist-Assisted Internet-Delivered Acceptance and Commitment Therapy in Primary Care for Adolescents With Anxiety: Naturalistic 12-Month Follow-Up Study
Source: JMIR Pediatr Parent. 2025 Jan 3;8:e59489. doi: 10.2196/59489 (PMC11748435; doi:10.2196/59489)
Supplement: Multimedia Appendix 1 [file pediatrics_v8i1e59489_app1.pdf]

Module title	Content	Summary
Module 1: How does anxiety work	• This is how you work in the program<br>• This is anxiety<br>• Nadia is worried about what others think<br>• Presentation by Nadia (Social Anxiety)<br>• Anna is afraid of her body's reactions<br>Presentation of Anna (Panic Disorder)<br>• Robin worries about everything that could happen<br>• Presentation by Robin (GAD)<br>• Carlos controls everything<br>• Presentation of Carlos (OCD)<br>• Dare to get in touch<br>• This program focuses on anxiety issues<br>• What are diagnoses?<br>• Are diagnoses a must?<br>• How does anxiety actually work?<br>• Trying to avoid the discomfort makes you miss out on life<br>• The human brain can see ahead in time<br>• The downside of the brain's ability<br>• Inner experiences become your monsters<br>• Everyone wants to avoid monsters<br>• What can you do instead of avoiding?<br>• About CBT<br>• Meet Moa<br>• Record how you feel during the treatment<br>• Registering Mission: Get a handle on your situation<br>Your situation<br>• Task: Plan your time with Angesthjälpen Ung<br>Time and place where I can work with Angesthjälpen Ung	The focus of the episode is to be introduced to the program and the way of working and to gain an understanding of what anxiety is, what triggers it, how trying to avoid it can have the opposite effect, as well as to start looking at one's own anxiety.<br><br>After the section, the client should be able to answer:<br>1. What is anxiety?<br>2. What does my anxiety look like?<br>3. What is the problem with constantly avoiding difficult situations?

	• Assignment during the week: map your anxiety<br>Map your anxiety	
Module 2: What's most important to you	• Now you are in focus<br>• Audio exercise: Imagine if all your anxiety went away<br>• To live the life you want<br>• Goals are like your sun<br>• Some know their goals - others don't<br>• Sound practice: The party<br>• The meaning of the exercise<br>• Your goals in different areas<br>• Nadia's goal<br>• Short-term and long-term goals<br>• Mission: your goals<br>• Hear Moa tell<br>• Monsters can stop you from moving towards your goals<br>• Example: Robin avoids the discomfort<br>• Example: Anna and the concert<br>• It will be a slip<br>• Examples of avoidances<br>• Mission during the week: Plan one of your short-term goals	The focus of the episode is to introduce short-term and long-term goals (values), and for the client to see their own, as a way to find motivation for long-term strategies rather than avoidance and control.<br>After the section, the client should be able to answer:<br>What are my short-term and long-term goals?<br>What is stopping me from moving towards my goals?
Module 3: Why Did You End Up Here?	• Time for the first tool<br>• Introducing the Toolbox<br>• How you can get stuck in a vicious circle<br>• The spotlight on situations and consequences<br>• Assignment: When do you experience the collision?<br>• Tool #1: The ABC checklist<br>• You can choose what you do, but not how you feel<br>• Clarification of the example in the previous video<br>• Assignment: Test directly<br>• Your short-term consequences<br>• The meaning of the tool ABC-check<br>• Carlos does the ABC check<br>• What can Carlos learn from his ABC test<br>• More examples of ABC checks	The focus of the episode is to understand the tool ABC-kollen (functional analysis) and in this way begin to reflect on how one's own anxiety problem is perpetuated and to get ideas about how to act in ways that work in the longer term.<br>After the section, the client should be able to answer:<br>1. How do you do an ABC check?<br>2. Why is it good to do the ABC check?

	• Anna does the ABC test on her fear of panic attacks<br>• Nadia does the ABC's of her anxiety about social situations<br>• Robin does the ABC's of a situation where a painful memory surfaces<br>• Common factors<br>• The action is guided by the short-term consequence<br>• Your monsters appear during "inner experience"<br>• Your goals can become "long-term consequences"<br>• Some tips about the ABC test<br>• You can start anywhere<br>• Assignment: test your ABC<br>• Tasks during the week: Do an<br>• ABC check during the week<br>• Scouting: What have you learned?	
Module 4: "I don't dare but I do it anyway"	• Welcome to module 4!<br>• Did you manage to get rid of your monsters?<br>• Example: Robin's attempt to get rid of his monsters goes badly<br>• The fight takes all the energy<br>• Example: Nadia avoids social contexts<br>• Avoidance leads away from what is important<br>• Fight and avoidance does not work in the long run<br>• Missions: Get a handle on your combat and evasions<br>• Mission: Draw your path<br>• What can you do instead?<br>• Tool 2: Directed action<br>• Take the monsters on your shoulder and move forward<br>• It's easy to get caught up in the struggle<br>• Secret escape from anxiety<br>• About security behaviors<br>• Moa's change<br>• Maybe you get stuck in the "but" trap?	The focus of the episode is to introduce the tool of directed action (exposure and acceptance) in contrast to avoidance and coping with anxiety as strategies. The aim is to start behaving more in line with your goals and practice accepting discomfort. After the section, the client should be able to answer:<br>1. What is directed action and how can it help me?

	• Assignment: Write down your targeted actions<br>• Anna plans targeted actions<br>• Mission for the week: Take a directed action AND have anxiety	
Module 5: Mind Zoom helps you move forward	• Do you get stuck in brooding?<br>• Thoughts that many can get stuck in<br>• How it can turn out for Nadia and Robin<br>• You have a thinking apparatus<br>• Your brain's task<br>• Explore how thoughts affect you<br>• Scouting: What have you learned from doing the lemon exercise?<br>• The thoughts get in the way<br>• The fight against the thought monsters takes all the energy<br>• Having thoughts, not being thoughts<br>• Thoughts vary<br>• Thought or interpretation<br>• How has it worked to get rid of the troublesome thoughts?<br>• Zoom in: Give the tank a name<br>• Hindering and helpful thoughts<br>• Carlos thinks he is bad at everything<br>• Assignment: Zoom in on a thought<br>• Mission: Zoom out from a thought<br>• Audio exercise: Zoom out<br>• Introduction to Soundcloud<br>• Mind zoom is used in conjunction with directed action<br>• Tasks during the week: Directed action and thought zoom<br>• Scouting: Reflection after practice	The focus of the episode is to introduce the mind zoom (defusion) tool. The purpose is to illustrate that what you think about yourself does not have to be true and that thoughts do not have to control your actions, regardless of what they contain.<br><br>After the section, the client should be able to answer:<br>1. What is mind zoom?<br>2. What should I do when thought monsters appear?
Episode 6: The Here and Now	• Explore your time machine<br>• Sound exercise: Practice conscious presence<br>• Carlos is listening to his favorite song<br>• Audio exercise: Shift focus	The focus of the episode is to introduce the tool of conscious presence in order to learn to experience what you experience without having judgmental thoughts about it and to be able

<table>
<tr><td></td><td>What is the meaning of mindfulness?
Directed action produces no effect without presence
Tool #4: Here's how
Nadia uses conscious presence in the dining room
Robin practices mindfulness as he plans the trip with his friends
Repetition of the steps
Sound practice: contact with the present
No one is present all the time:
Become an observer
Anna has a lot of inward focus
The important thing is the ability to shift focus
Sound Practice: The Key to Presence
When the going gets tough
Mission during the week: To be here and now
Part 1: Contact with the present
Part 2: Conscious presence in everyday life
My training sessions</td><td>to practice your ability to choose what you want to focus your attention on.
After the section, the client should be able to answer:
1. What is mindful presence?
2. When can you use mindful presence?</td></tr>
<tr><td>Episode 7: Walk Towards Your Sun</td><td>Get better control of your tools
Assignment: Check your goals from module 2
Compare your goals then and now
Your four tools for living life despite anxiety
How can you use the tools when things get extra difficult?
Sound practice
Example: Robin
Example: Anna
Moa talks about what she learned
Mission during the week: Go towards your sun
Your targeted actions
Jumping into life
Assignment during the week: The collage</td><td>The focus of the episode is to learn more about how the four tools can be integrated and how to use them when the going gets tough.

After the section, the client should be able to answer:
1. When can I use the various tools?
2. How can I use the tools when it gets extra difficult?</td></tr>
</table>

Episode 8: Make a plan for the future	• Welcome to the last module in the Anxiety School<br>• About not feeling ready<br>• Show compassion to yourself<br>• Assignment: Write down your chess rules<br>• When difficulties come<br>• Example: Anna faces a difficulty<br>• Moa talks about the future<br>• Be prepared for setbacks<br>• Mission: My plan for the future	The focus of the episode is to summarize lessons learned from treatment and make a plan to prevent post-treatment relapse. After the section, the client should be able to answer:<br>1. How can I continue working on my own after the Anxiety School?<br>2. How can I deal with setbacks in the future?
