# Supplementary material for: The Impact of Parental Support on Adherence to Therapist-Assisted Internet-Delivered Acceptance and Commitment Therapy in Primary Care for Adolescents With Anxiety: Naturalistic 12-Month Follow-Up Study
Source: JMIR Pediatr Parent. 2025 Jan 3;8:e59489. doi: 10.2196/59489 (PMC11748435; doi:10.2196/59489)
Supplement: Multimedia Appendix 2 [file pediatrics_v8i1e59489_app2.pdf]

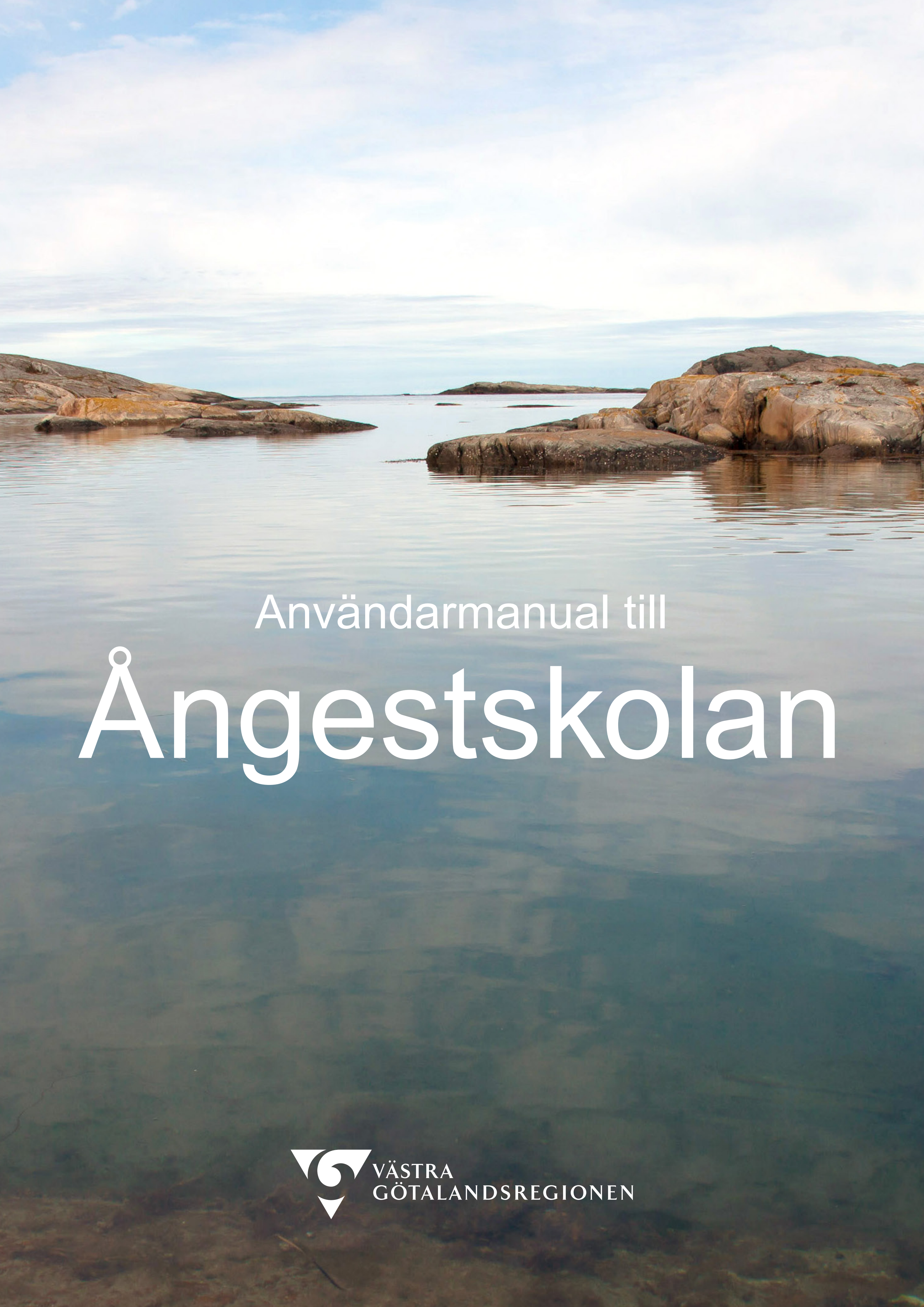

Användarmanual till

Ångestskolan

VÄSTRA GÖTALANDSREGIONEN

Det här är en kort information om vad ångest är och olika sätt att hantera den. Materialet är framtaget av BUP, men kan användas även i primärvården och andra verksamheter där man möter barn och ungdomar med ångest.

Innehåll

Samtal 1 4
- Det evolutionära perspektivet 4
- De tre "F-en"; fight, flight och freeze. 5
- Kroppsliga reaktioner 5
- Skarpa larm och falska larm 5
- Ångestkurvan 6

Samtal 2 7
- Behaglig tanke 7
- Obehaglig tanke 7
- Automatiska tankar 7
- Ångesthantering 7
- Andning 7
- Avslappning 8
- Avledning 8
- Utåtfokusering 8
- Medveten närvaro 8

Samtal 3 9
- Kartläggning av patientens ångest 9
- Skapa en hierarki 9
- Fördelar och nackdelar med din ångest 9

Referenslista 10

Mellan samtalen kommer du och dina viktiga vuxna få uppgifter att träna på och övningar att prova.
Ångestskolan är en så kallad psykoedukativ insats som patient och deras viktigaste vuxna kan erbjudas inom BUP. Den kan erbjudas inom såväl öppen, sluten och dagvård. De behandlare som ger ångestskolan ska ha åtminstone grundläggande kunskaper i KBT samt goda kunskaper och erfarenhet av psykiatrisk ångestproblematik. Det går att ge ångestskolan som ensam behandlare men det finns fördelar om det är möjligt med två behandlare. På BUP-akutenhet i Göteborg ges ångestskola vanligtvis av psykolog och sjuksköterska ihop.
Ångestskolan är alltså ingen behandling eller utredning och kan inte fungera som ett substitut för något av det. Däremot är ångestskolan en bra och inte sällan nödvändig introduktion till bägge.

Foton från fotoakuten.se

Denna behandlarmanual får ses som en stomme och inte en komplett redovisning av alla tänkbara exempel, strategiförslag eller interventioner. Varje ångestskola ska skräddarsys efter patientens ålder, behov förutsättningar och situation. Det är dock viktigt att alla behandlare följer stommen och berör alla punkter som tas upp. Punkter hänger ihop och behövs för att patient och dess viktiga vuxna ska förstå och uppleva sig förstådda.
Ångestskolan är framtagen på korttidsvårdsavdelningen Slottis där den ges till alla patienter och deras viktiga vuxna. Det har tidigare inte funnits en manual utan en "kom ihåg-lista" – se bilaga. Det har från olika delar framkommit önskemål om en behandlarmanual. Detta är det första förslaget på en sådan.

Ångestskolan går inte igenom patientens sömn-, kost och motionsvanor. Allt detta är såklart viktiga delar i all form av psykisk ohälsa. Det berörs dock inte direkt i just ångestskolan men kan givetvis nämnas.

Ångestskolan ges till barn och ungdomar tillsammans med deras viktigaste anhöriga som vanligtvis är föräldrarna. Den består av tre tillfällen på cirka 1-1,5 timme per gång. Eftersom det är relativt långa träffar är det lämpligt att erbjuda te eller kaffe och att ha tillgång till vatten att dricka i rummet. Det ger även en avslappnad inramning som många patienter och deltagare enligt erfarenheten uppskattar.

Ångestskolan är en undervisning i vad ångest är från ett psykobiologiskt perspektiv och en introduktion till hur ångest kan hanteras från kognitivt-beteendeterapeutiskt synsätt. Den ska vara informativ men ges i aktiv dialog med ungdomen och dess viktiga vuxna. Det är viktigt att ungdomen får komma med egna exempel, synpunkter och frågor. Det samma gäller föräldrarna.

Behandlaren/behandlarna ska använda whiteboard eller papper för att illustrera det som förklaras.

Ångestskolan ska handla om ångest men inte ge det. Det ska vara tryggt och givande för ungdom och vuxna att delta. Behandlare ska vara lyhörd för patienten och anhörigas synpunkter och vara noggranna med att avsluta varje tillfälle med en utvärdering – muntlig eller skriftlig.

En väl utförd ångestskola ger patient och viktiga en ökad förståelse av ångest och dess mekanismer. Dessutom ger den behandlare viktig information om patientens ångestproblematik. Informationen är också ett bra underlag för fortsatt behandling och även indikation på om det behövs utredning eller fördjupad anamnes.

Samtal 1

En bra inledning är att fråga vad patienten och dess anhöriga vet om ångest. Ganska ofta vet patienten mer än föräldrarna.

Det evolutionära perspektivet

Här ska en kort beskrivning ges av det psykobiologiska förklaringssättet av ångest. Det är viktigt att stämma av med patienten och anhöriga vad de själva känner igen sig i och inte. Syftet med denna del är att tydliggöra att ångest inte är onormalt eller farligt även om det är mycket obehagligt och kan vara skrämmande. Ångest är samma sak som oro, rädsla, stress, skräck, nervositet eller att vara blyg. Det är en helt naturlig känsla som alla kan känna igen sig i (Chilled, ungdomsmanual) .

Inom evolutionspsykologin ser man ångest som det som hjälpte våra förfäder att överleva i en primitiv miljö (Leahy "ångestfri" 2009). Under stenåldern omgavs människan att ständiga hot som svält, farliga djur, utstötning ur stammen och väderomställningar. För att vara förberedd att klarar dessa svårigheter gällde det att oroa sig. Om man inte oroade sig för om maten skulle räcka över vintern, aktade sig för farliga djur eller såg till att fungera väl i gruppen var risken stor att man inte överlevde. Oro och ångest var naturens sätt att få oss att vara försiktiga och att skärpa våra sinnen (Ångestens ansikte-ASS-www.angestsyndromsallskapet.com). Oron och rädslan får att reagera på faror, som ett larmsystem.

Det är bra att anpassa illustrerande exempel utifrån patientens egen ångestproblematik. En person som har generell ångestproblematik kan till exempel jämföras med en samlare som på stenåldern oroade sig över mattillgång, väder, farliga djur, hotande grannstammar och så vidare. En person med tvångsproblematik kan illustreras med hur ordning och reda, upprepning kan vara nödvändigt för att ha matlager över vintern. Det psykobiologiska perspektivet kan appliceras på väldigt många olika ångestsyndrom: paniksyndrom, GAD, OCD, PTSD, social fobi och specifik fobi.
Det ska framgå att ångesten ur ett evolutionsperspektiv har hjälpt människan som art att överleva.

Ett exempel som annars går att använda är hur en grupp jägare är ute på savannen för 10 000-tals år sedan och anar rörelser i närliggande dunge. Då beskrivs hur uppmärksamheten skärps hos jägarna och hur kroppen förbereder sig på handling.

I dagens svenska samhälle utsätt människan inte för samma slags hot som stenåldersmänniskan hade över sig. Vi har dock kvar samma biologiska och psykiska reaktionsmönster. Många av de ångestsyndrom som plågar nutidsmänniskan har sitt ursprung i de rädslor som programmerats in i oss under evolutionens gång.

Det passar ofta bra att fråga om patienten eller anhöriga har egna exempel.

De tre ”F-en”; fight, flight och freeze.

Eftersom begreppet är väl etablerat på engelska inom svensk behandling beskrivs de så här. Det går givetvis alldeles utmärkt att översätta till att kämpa, fly och frysa till.
De vanligaste reaktionsmönstren när ångest väckts brukar kallas ”fight, flight eller freeze”. Ur ett evolutionsperspektiv förklaras dessa reaktioner med att du antingen kan försvara dig mot faran, fly från den eller i extrema fall spela död.
Vilket reaktionsmönster som patienten känner igen sig i kan vara viktigt diagnostisk information.

Försvarsmekanismen fight

Är att hantera ångestpåslaget genom att gå till attack. I exemplet från stenåldern kan det vara att gå till anfall mot rovdjur som närmar sig.
Översatt till en situation patienten kan känna igen sig i kan man beskriva ”fight” som försvarmekanism visa sig som ilska, skrik, våldsamt beteende eller annat utagerande beteende.

Försvarsmekanismen flight

Innebär att man flyr från det som upplevs som farligt-till exempel en rörelse i skogsgunge. Relevanta exempel för patienten kan vara att inte gå till skolan för att man är nervös för att hålla föredrag eller aldrig åka buss och spårvagn på grund av bacillskräck.

Försvarmekanismen ”freeze” illusteras bra av hur gnagardjur ute på ett slätt kan spela döda när de ser skuggan av en rovfågel på marken. Ett annat exempel är att stora däggdjur som delfiner kan spela döda upp de fångas i fiskenät.
Om en patient beskriver att den känner igen sig i denna försvarmekanism är det lämpligt att vid senare tillfälle undersöka dissositiva symptom hos patienten.
Här är det igen viktigt att få höra patientens egna exempel. Ofta känner patienten igen sig mest i ett av reaktionsmönstren men många känner igen sig i flera.

Kroppsliga reaktioner

Det som händer i kroppen när man får ångest är bland annat hjärtklappning, grundare andning och svettningar. Muntorrhet, spända muskler, domningar, stickningar, illamående, klump i halsen, andnöd och yrsel är också vanligt.
Man blir extremt uppmärksam på det som väckt ångesten.

Skarpa larm och falska larm

I dagens samhälle utsätts man sällan för direkt dödsfara på samma sätt som stenåldersmänniskan.
Om en person går ut i en gata utan att titta efter bilar och en bil kommer rusande i hög hastighet i kommer oro, rädslan slå till. Personen kommer att fokusera på bilen och kasta sig upp på trottoaren. I detta fall har personen reagerat på ett så kallat skarp larm. När ångesten blir till ett problem är när den slår till på så kallade falska larm. Det är situationer eller omständigheter som inte är farliga. Om personen helt slutar gå över gator av rädsla över att kunna bli påkörd så reagerar hen på ett falsk larm eftersom det inte är farligt att gå över alla gator.

Kroppen reagerar på både falska och skarpa larm innan man vet om faran är verklig eller inte. Dessutom är ångest något man snabbt kan lära sig att känna. Om man nästan blivit påkörd av en bil kommer de flesta människor vara mer nogranna med att se sig för nästa gång de går över gatan. De har lärt sig att det kan vara farligt att slarva med att se åt höger och vänster.

Tyvärr är inte ångestpåslaget logiskt eller rationellt-det kan slå på för saker som inte är ett dugg farliga. Här är det bra om deltagarna kan ge egna exempel på situationer som ger dom ångest utan att de egentligen är farliga.

Ångestkurvan

Syftet är bland annat att beskriva hur en kraftig ångestreaktion är i sitt initiala skede, vad som sker på kort och lång sikt om man flyr från den skrämmande situationen samt vad som sker om ångesten istället får ha sitt naturliga förlopp (Öst, 2010). Det är det viktigt att förklara att ångesten går över och inte farlig. Det är också viktigt att prata om att ångest är mycket obehagligt och något alla människor helst undviker att ha. Exempel som många kan känna igen sig i är när man skulle lära sig att simma, cykla eller börja övningsköra. I början känns det jätteoroligt och skrämmande men med till och övning blir oron svagare för att till sist klinga av helt. Om man däremot inte fortsätter träna på att cykla efter att ha ramlat en gång är det sannolikt att man utvecklar en rädsla för att försöka igen.

Nästa del är att beskriva kortsiktiga och långsiktiga konsekvenser att att fly från eller att undvika situationen som väcker ångest. Kortsiktigt går ångest ner snabbt. Långsiktiga konsekvenser blir att samma situation blir ännu mer skrämmande nästa gång eftersom man har lärt sig att man måste fly för att ångesten ska gå ner.
Det är viktigt att resonera en del med deltagarna om kortsiktiga men framförallt långsiktiga konsekvenser.

En del patienter känner inte igen sig väl i den högintensiva ångesten utan har en mer lågfrekevent, ständigt närvarande oro. Ofta finns det dock situationer där den stegras som patienten undviker, flyr eller utstår med stort lidande. I det sistnämnda fallet kanske personen stannar kvar i i situationen men gör det genom olika säkerhetsbeteenden som att räkna tyst för sig själv, inte säga något (Öst, red 2010). Dessa exempel kan man återkomma till under delen som handlar om hur tanke, känsla och handling hänger ihop.

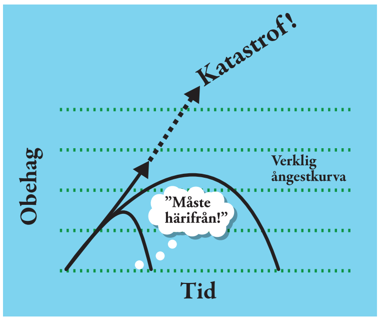

Ångestkurvan. Den tidigare rädsloreaktionen avtar
eller förlorar i styrka (psykologiguiden, psykologforbundet.se).

Samtal 2

Samspel mellan tanke, känsla och handling

Syftet är att beskriva hur tanke, känsla och handling hänger ihop och påverkar varandra hos alla människor och att det går att påverka sina tankar och sina handlingar i högre grad än känslor. Det är viktigt att rita upp exempel och gärna exempel både från patient och anhöriga.

Behaglig tanke

Syftet är att ge exempel på hur en behaglig tanke får det att kännas i kroppen och hur det kan påverka beteende. Det är givetvis önskvärd med patientens egna exempel.

Obehaglig tanke

Syftet är att ge exempel på hur en obehaglig tanke får det att kännas i kroppen och hur det kan påverka beteende. Det är givetvis önskvärd med patientens egna exempel.

Automatiska tankar

Syftet är att förklara att denna typ av tankar dyker upp oreflekterat och vanemässigt och har därmed i många fall dålig koppling till den verklighet som de refererar till (www.psykologiguiden.se). Om tankarna som dyker upp i medvetandet är ifrågasättande och nedvärderande ger de upphov till omotiverad ångest och depressiva reaktioner. De väcker känslor av otillräcklighet och hopplöshet som i sin tur förstärker de automatiska tankarna i en ond cirkel.

Ångesthantering

Syftet är att beskriva olika sätt att hantera ångest på. Detta är ett bra tillfälle att fråga vilka strategier patienten redan provat och vilka som fungerat och inte. Det ska bli tydligt att man behöver träna på strategier när man inte har stark ångest för att kunna använda dem när ångesten är värre. Det ska också vara tydligt att det är nödvändigt att öva på strategierna för att kunna använda dem rätt och väl under ångest. Behandlarna ska också förklara att varje individ kan ha personlig strategier som fungerar och inte är någon av de som går igenom på ångestskolan.
I behandling av ångestproblematik finns det en risk av hanteringsstrategier utvecklas till såkallade säkerhetsbeteenden (Öst et al 2010).
Ångestkolan är dock ingen behandling och beskriver djupandning och tillämpad avslappning eftersom det kan fungera dämpande vid ångest. Det är ofta nödvändigt att patienten har ett antal strategier för av våga exponera sig för sin ångest (Öst et al 2010).
När ångestskolan är avslutat får de behandlare som fortsätter träffa familjen vara uppmärksamma om patienten använder strategierna som ett säkerhetsbeteende.

Andning

Det är vanligt som redan beskrivits vanligt att överandas eller hyperventilera när man blir rädd eller ängslig. Ett sätt att komma till rätta med det är att träna på så kallad magandning (Öst red 2010).
Här gås igenom hur en lugn djupandning påverkar kroppen såsom muskelavspänning

och syresättning av blodet. Patienten och föräldrarna får pröva att andas lugnt och djupt och notera hur det påverkar kroppen. En metafor som är användbar är att patienten har en ballong i magen som med djupa andetag fylls med luft och som sedan i en utandning pyser ut luft och ballongen blir liten igen.

Avslappning

Genom att träna avslappning kan du lära dig att bättre kontrollera ångesten och dämpa kroppsliga tecken som muskelspänning och huvudvärk.
En väldigt enkel metafor att prova under besöket är att tänka sig att man är först ett okokt och sedan ett kokt spagettistrå (TF-CBT 2010, Öst red 2010)
Man kan också tipsa deltagarna om att det på apoteket kan du köpa CD-skivor med avslappningsövningar (vårdguiden.se). Det finns en en hel del appar som går att köpa om man har en smartphone.
Det som ska framgå för patienten är skillnaden mellan en spänd och avslappnad kropp.

Avledning

Denna strategi handlar just om vad som kan fungera för patient som avledning från ångesten. Många tycker att musik, internet och telefonsamtal fungerar som avledare. Behandlare och deltagare kan tillsammans fundera kring vad patienten kan pröva och då förorda att göra något socialt, kreativt eller fysiskt.

Utåtfokusering

Denna strategi handlar om att fokusera på något i omgivning. Syftet att flytta fokus från upplevelsen av ångest till något annat.
Exempel på övningar att pröva är:
Patienten ska säga fem saker den ser, känner och hör i rummet. Det går utmärkt att upprepa saker. Behandlaren ska visa hur övning går till. Efter att ha sagt fem av varje säger man fyra,tre, två och en. Denna typ av teknik kan efter övning lätta patientens ångest och möjlighet att gå emot eller ta fokus från ångesten.
En annan välbeprövad teknik kallas att ”surfa på vågen”. Den handlar kort uttryckt om att när ångesten kommer få en mental bild av hur ångesten kommer som en våg och att man själv surfar på vågen. Som alla vågor når den en maxhöjd för att sedan brytas mot stranden. Detaljerad beskrivning av övningen finns bland annat i ungdomsmanualen ”Chilled”.

Medveten närvaro

Denna teknik handlar väldigt enkelt och kort uttryckt om att iaktta sina egna upplevelser direkt utan att döma eller värdera (vårdguiden.se).
Exempel på övningar är att äta äpplen och nogrannt registera smak, textur, ljud och så vidare. Metoden för mindfulness är enkel men kräver regelbunden övning för att ge bästa resultat. Du kan naturligtvis ha glädje av tankarna bakom mindfulness även om du inte har möjlighet att ägna metoden så mycket tid. Du kan öva på närvaro och att uppleva vad som händer runt omkring dig när som helst. Tänk på och känn dofterna när du lagar mat, se grönsakernas färgnyanser och upplev smakerna i maten.

Samtal 3

Kartläggning av patientens ångest

Här undersöker man ihop med patienten när patienten får ångest och vilka situationer som är ångestväckande för patienten. Man kartlägger också noggrann hur ångesten upplevs för patienten. Här används whiteboard eller papper och penna.

Skapa en hierarki

Patientens ångestproblem delas upp i en ångesthierarki, som innefattar de situationer som är ångestväckande för patienten, alltifrån sådana som väcker lätt ångest till sådana som väcker den allra starkaste ångesten (psykologiguiden.se 2013). I själva behandlingen börjar man med de ångestsituationer som finns längst ner i hierarkin och fortsätter uppåt allteftersom patienten klarar av de lättare situationerna. I ångestskolan påbörjar man inte en behandling men förklarar att det är lämpligast att börja med det lättaste, precis som att börja med stöd eller stång jämfört med att att försöka börja cykla helt själv.
Det kan vara till hjälp att använda en skala som till exempel 0-10. Vid noll upplevs ingen oro, fem medelstark vid tio är oron så stark som den kan bli.

Fördelar och nackdelar med din ångest

Även om det är plågsamt att ha ångest kan det även ge fördelar. Till exempel kan det vara så att man att man slipper en del krav eller förväntningar. Här ska man på ett förstående men tydligt sätt prata om både vilka fördelar och nackdelar ångesten ger patienten.

Referenslista

Vårdguiden 1177

OSS-ångestsyndromsällskapet

Psykologiguiden

TF-CBT

”Chilled” Manual för behandling av ångest för tonåringar

”KBT inom barn och ungdomspsykiatrin” Lars-Göran Öst red (2010)

”Ångestfri – bli av rädslor och fobier” av Robert L. Leahy (2010)

”Kognitiv psykoterapi i klinisk tillämpning”Arthur Freeman et al (1994)

”Clinicial practice of cognitive therapy with children and adolescents” Frieberg et al (2002)
